# The cohesin complex prevents Myc-induced replication stress

**DOI:** 10.1038/cddis.2017.345

**Published:** 2017-07-27

**Authors:** Sara Rohban, Aurora Cerutti, Marco J Morelli, Fabrizio d'Adda di Fagagna, Stefano Campaner

**Affiliations:** 1 Center for Genomic Science of IIT@SEMM, Fondazione Istituto Italiano di Tecnologia (IIT), Via Adamello 16, 20139 Milan, Italy; 2 IFOM Foundation-FIRC Institute of Molecular Oncology Foundation, Milan 20139, Italy; 3 Istituto di Genetica Molecolare, CNR – Consiglio Nazionale delle Ricerche, Pavia 27100, Italy

## Abstract

The cohesin complex is mutated in cancer and in a number of rare syndromes collectively known as Cohesinopathies. In the latter case, cohesin deficiencies have been linked to transcriptional alterations affecting Myc and its target genes. Here, we set out to understand to what extent the role of cohesins in controlling cell cycle is dependent on Myc expression and activity. Inactivation of the cohesin complex by silencing the RAD21 subunit led to cell cycle arrest due to both transcriptional impairment of Myc target genes and alterations of replication forks, which were fewer and preferentially unidirectional. Ectopic activation of Myc in RAD21 depleted cells rescued Myc-dependent transcription and promoted S-phase entry but failed to sustain S-phase progression due to a strong replicative stress response, which was associated to a robust DNA damage response, DNA damage checkpoint activation and synthetic lethality. Thus, the cohesin complex is dispensable for Myc-dependent transcription but essential to prevent Myc-induced replicative stress. This suggests the presence of a feed-forward regulatory loop where cohesins by regulating Myc level control S-phase entry and prevent replicative stress.

The cohesin complex is composed of the core subunits SMC3, SMC1A, RAD21, SA1/STAG1 or SA2/STAG2 and several other accessory proteins that regulate its loading and unloading onto chromatin.^1^ This complex is believed to ensure physical cohesion between sister chromatids by forming a ring-shaped structure that embraces DNA strands.^2, 3, 4^ Owing to its biochemical function, the cohesin complex has been implicated in the control of diverse processes requiring sister chromatids association and long distance DNA contacts, ranging from the control of chromosome segregation, DNA repair, DNA replication and gene transcription.^5^ In particular, in recent years a rising number of evidences highlighted a role of the cohesin complex in determining nuclear architecture and the tridimensional organization of chromatin compartments.^6, 7^ Indeed, the cohesin complex, in association with CTCF, is required for transcriptional insulation in vertebrate cells,^8^ but also cohesins can associate with a number of active promoters in a CTCF-independent way,^9, 10^ to regulate gene transcription, in part by favoring higher order chromatin interactions to allow promoter–enhancer contacts.^11^

The ability of cohesins to regulate transcription is essential to maintain pluripotency in ES cells^12^ and to support transcriptional programs during development.^13^ Cohesin mutations have been identified in a series of syndromes collectively known as cohesinopathies.^14^ The best characterized member of these family of disorders is the Cornelia de Lange syndrome (CdLS). While cohesin mutations identified in CdLS do not seem to affect DNA cohesion *per se*, they seem to affect transcription,^15, 16, 17^ an observation confirmed by studies in model systems.^18, 19, 20, 21^ In particular, the cellular defects due to disease-associated mutations have been linked to c-Myc downregulation.^13, 15, 22, 23^

c-Myc (hereafter Myc) is a transcription factor of the Helix-loop-helix leucine zipper family, which in normal cells is expressed in response to growth factors and mitogenic signals as part of the immediate early genes program.^24^ As a transcription factor, Myc forms a heterodimeric complex with its partner Max to regulate the expression of several genes implicated in cell growth, cell cycle control, cell differentiation and identity.^25^

A number of evidences link cohesin’s function to Myc activity: (i) expression of Myc and cohesins is positively correlated through evolution and several evidences highlight the requirement of cohesins to support the expression of both Myc and its target genes^13, 15, 22, 23^; (ii) recent genome-wide studies have highlighted the presence of genomic sites, where the cohesin complex and Myc seem to have a relevant role in co-regulating transcription^26^; (iii) Myc over-expression has been shown to be synthetic lethal with loss of RAD21.^27^

In this manuscript we address the relationship between the cohesin complex and Myc with a particular focus on their respective ability to control both transcription and DNA replication. Our data show that RAD21 silencing affected the expression of a subset of genes related to cell cycle control, many of which are Myc targets. Loss of Myc-dependent transcription was rescued by ectopic activation of Myc, thus suggesting that elevating Myc levels can bypass RAD21 dependency. On the other hand, while forced activation of Myc was sufficient to restore transcription of its target genes and promoted S-phase entry in RAD21-deficient cells, it also triggered replicative stress (RS). This was due to defects in replication fork initiation and progression, which led to the development of a cytotoxic DNA damage response (DDR) followed by the engagement of the DNA damage checkpoint. Overall, these data suggest that while RAD21 may be dispensable for Myc-induced transcription and S-phase entry, it is absolutely required for faithful DNA replication. This provides a rationale to understand how cohesins regulate gene expression and cell cycle progression, and unveils a peculiar role of RAD21 in preventing Myc-induced RS.

## Results

### Ectopic activation of Myc rescues transcription of its target genes and cell cycle progression in RAD21-depleted cells

**Figure 1:**
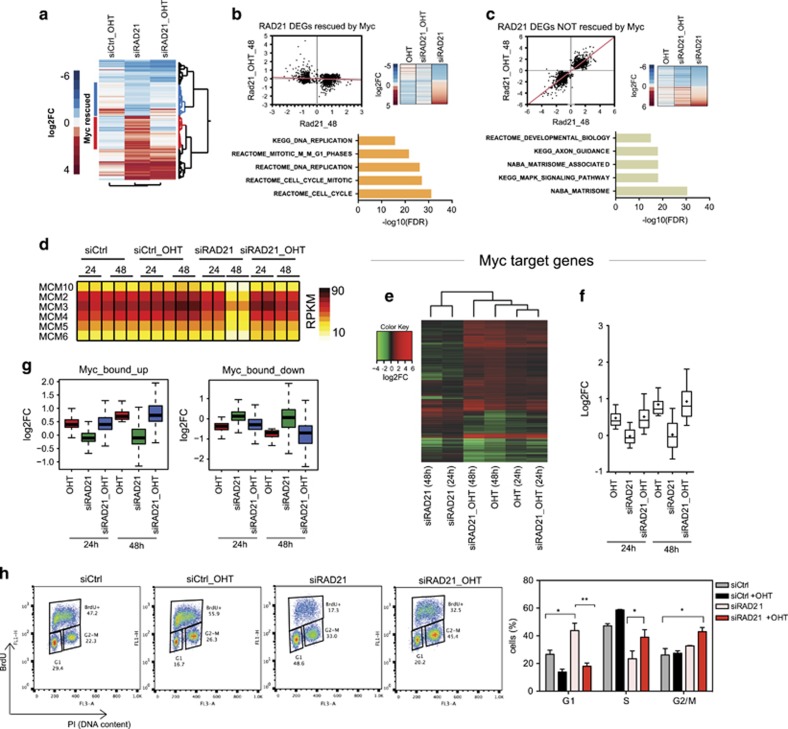
Transcriptional and cell cycle alterations due to loss of RAD21 are partially rescued by MycER. U2OS-MycER cells were transiently transfected with siRNAs targeting either RAD21 (siRAD21) or Renilla Luciferase (siCtrl) and were simultaneously treated with either OHT (to activate MycER) or ethanol (as mock activation). (**a**) Unsupervised hierarchical clustering of RAD21-DEGs based on the fold change in expression (Log2FC=log2 fold change) determined at 48 h post-RAD21 silencing. Clusters of upregulated and downregulated gene sets rescued by Myc activation in RAD21 depleted cells are shown by red and blue bars, on the left side of the heatmap. (**b**) RAD21-DEGs rescued by MycER activation: on the left, scatter plot showing gene expression upon RAD21 silencing compared to RAD21 silencing and MycER activation, red line is the fit line (linear regression); on the right, ranked heat-map of expression reported as Log2FC; bottom, top 5 gene-sets identified by GSEA analysis. (**c**) RAD21-DEGs not rescued by MycER activation. Panels as in (**b**). (**d**) Heat map of expression level of MCM gene sets based on expression levels (RPKM). (**e**) Clustered heat map showing the expression of Myc-target genes, reported as Log2FC relative to control cells (siCtrl). Time indicates hours after transfection/treatment. Red and green show up- and downegulated genes, respectively. (**f**) Box plot showing the log2 fold change values of genes upregulated by MycER, as in (**e**). (**g)** Box plots of the log2 fold change of Myc deregulated genes that are also bound by Myc at their promoter. Left panel: upregulated genes; Right panel: downregulated genes. (**h**) Cell cycle distribution of transfected cells at 48 h after siRNA transfection and OHT treatment as determined by BrdU pulse-labeling (30 min) and FACS analysis. Bar chart shows the fraction of cells in Go/G1, S and G2/M. Data are presented as mean±standard deviation of three independent samples (two-way ANOVA+Tukey’s multiple comparisons test, *P<0.05; **P<0.01; ***P<0.001; ****P<0.0001)

To investigate the possible interplay between Myc and the cohesin complex in transcriptional regulation, we profiled gene expression by RNA-seq in exponentially growing U2OS cells. RAD21 knockdown led to a progressive alteration of the transcriptome, with around 4000 genes differentially expressed (RAD21-DEGs) at 48 h of silencing (Figure 1a and Supplementary Figure 1a). Gene set enrichment analysis (GSEA) revealed an enrichment of cell cycle genes and Myc target genes among the genes that were downregulated upon RAD21 silencing (Supplementary Figure 1b and Supplementary Table 1). Since also Myc was downregulated, both at the mRNA and at the protein level (Supplementary Figure 1c,d), we asked whether its enforced activation could rescue transcription. We engineered U2OS cells to express MycER, a chimeric fusion of the Myc protein with the estrogen receptor that is activated by 4-hydroxytamoxifen (OHT).^28^ MycER levels were not affected by RAD21 silencing (Supplementary Figure 1d). We then performed RNA-seq analysis on U2OS-MycER cells, upon silencing of RAD21 and MycER activation. Hierarchical clustering of RAD21-DEGs revealed the presence of two clusters of genes that were rescued by MycER (clusters marked in red and blue, Figures 1a and b). GSEA analysis revealed that rescued genes were linked to cell cycle control and cellular proliferation (Figure 1b,Supplementary Figures 1e,f and Supplementary Table 2), while all the other RAD21-DEGs (i.e. not rescued by Myc) were ontologically linked to extracellular matrix organization/signal transduction (Figure 1c, Supplementary Figure 1g and Supplementary Table 2). Among the MycER rescued, there were genes directly implicated in the control of DNA synthesis such as MCMs (Figure 1d and Supplementary Figure 1e). Overall, MycER regulated genes, although affected by RAD21 knockdown in mock-treated cells, were largely restored by OHT (Figures 1e and f and Supplementary Table 3). This was confirmed when considering direct Myc targets (genes with promoters bound by MycER in U2OS cells^29^) (Figure 1g,Supplementary Table 4). This suggests that RAD21 can control cell cycle and cell proliferative programs by regulating Myc levels and indicates that transcription of Myc target genes does not require RAD21.

Given the transcriptional alteration of cell cycle genes observed upon RAD21 silencing, we also profiled cell cycle by FACS. While there was no significant change in the cell cycle profile at 24 h post RAD21 silencing (data not shown), at later time points (i.e. 48 h) we observed a remarkable increase in the G0/G1 population and a concomitant reduction of cells undergoing DNA synthesis (Figure 1h). MycER activation partially rescued these cell cycle defects (Figure 1g). Thus, ectopic activation of Myc restored both Myc-dependent transcription and cell cycle progression in RAD21-depleted cells.

### Prolonged Myc activation in RAD21-depleted cells triggers DNA damage response and synthetic lethality

**Figure 2:**
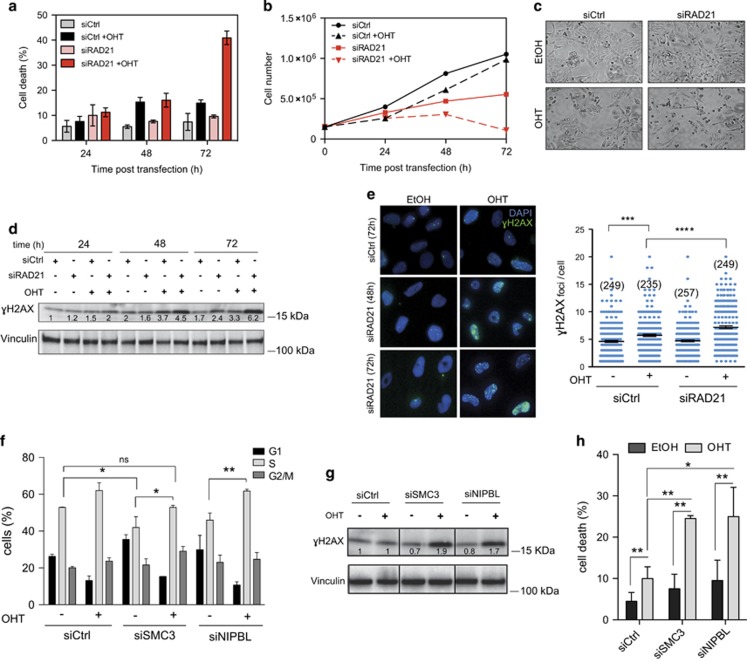
RAD21 limits DDR and promotes cell survival upon ectopic activation of Myc. U2OS-MycER cells transfected with siRNAs against either RAD21 or Rluc (siCtrl) and simultaneously treated with OHT or ethanol (EtOH). (**a**) Bar graph of the percentage of dead cells determined by trypan blue staining at 24, 48 and 72 h post treatment. Data are presented as mean±standard deviation (*n*=3). (**b**) Growth curve, as in (**a**). (**c**) Representative pictures of cells at 72 h post transfection. (**d**) Western blot analysis showing phosphorylation of H2AX (γH2AX) in the Myc-activated cells depleted of RAD21. Vinculin was used as a loading control. Numbers are the normalized intensity of the γH2AX bands. (**e**) Left: Representative images of γH2AX foci (green) in the transfected cells at the indicated times post treatment. Nuclei were counterstained with DAPI (blue). Right: dot-plot of the number of γH2AX foci/cell counted at 48 h post siRNA transfection/treatment. Total number of cells is shown in parentheses. Significant differences are indicated by asterisk (*t*-test: ***P<0.001; ****P<0.0001). (**f**) Bar plot of the cell cycle analysis performed by FACS on BrdU pulse-labeled cells. U2OS-MycER cells transfected with siRNAs against either SMC3, NIPBL or siCtrl were analyzed at 48 h post silencing/OHT treatment. (**g**) Western blot analysis, as in (**f**). (**h**) Percentage of the dead cells at 48 h, measured by trypan blue staining. (**f, h**) Data are presented as mean±standard deviation (two-way ANOVA+Tukey’s multiple comparisons test, *P<0.05; **P<0.01)

While Myc rescued both the cell cycle and transcription, we observed that its prolonged activation led to strong lethality in RAD21-depleted cells (Figures 2a–c and Supplementary Figure 2a), confirming published observations.^27^ Given the role of cohesins in DNA replication and genome stability, we wondered whether, similarly to what previously observed with components of the replication checkpoint,^30^ depletion of cohesins might exacerbate Myc-induced DDR and thus account for the synthetic lethal phenotype observed. DDR was assessed by measuring the level of the DNA damage marker γH2AX by western-blotting. While MycER activation led to the expected accumulation of γH2AX, this was further enhanced by RAD21 knockdown (Figure 2d and Supplementary Figure 2b). Single cell immunofluorescence analysis confirmed that activation of MycER in RAD21-depleted cells led to a significant increase in the number of γH2AX-positive cells (Figure 2e). While in RAD21-silenced cells DDR approached its maximum at 48 h of MycER activation (Figure 2d), cell death peaked at later time points (72 h) thus suggesting that DDR preceded synthetic lethality. As observed for RAD21, silencing of SMC3 and NIPBL, two subunits of the cohesin complex, also led to cell cycle arrest, which was rescued by MycER activation (Figure 2f). Accordingly, prolonged Myc activation in both SMC3 and NIPBL silenced cells triggered DDR and cell death (Figures 2g and h,Supplementary Figure 3). Altogether, these results are similar to what observed upon Rad21 silencing, albeit less strong. Whether this reflects cohesion independent function of RAD21 is a matter that will require further assessment.

### Myc activation rescues S-phase entry in RAD21-depleted cells but provokes replicative stress

**Figure 3:**
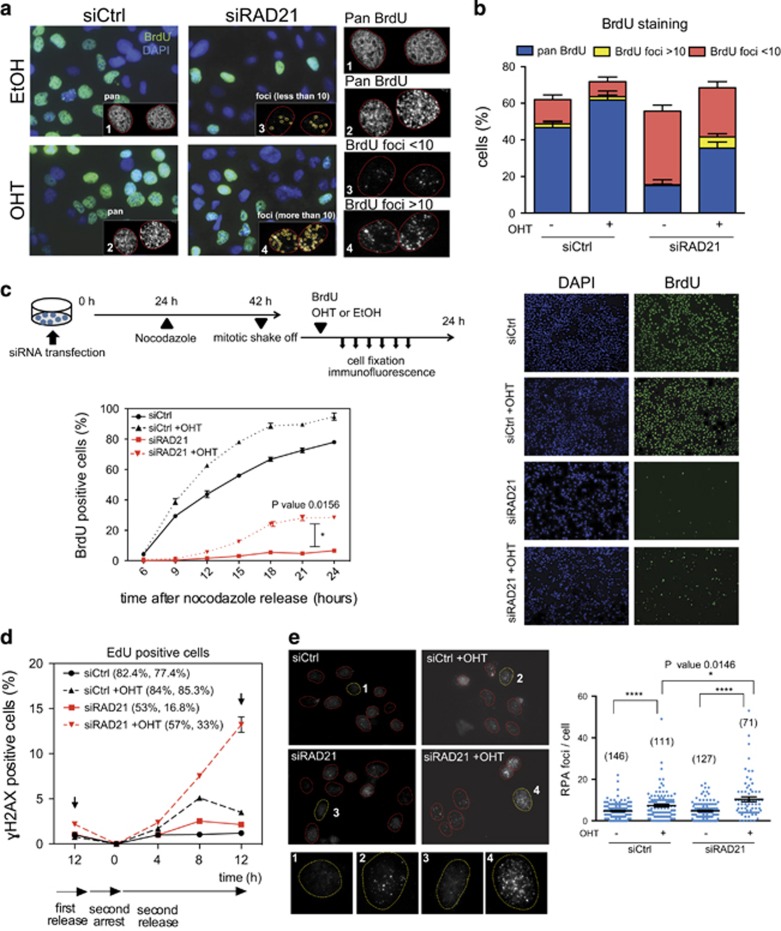
Myc-induced S-phase entry leads to replicative stress in RAD21-depleted cells. U2OS-MycER cells were transfected with siCtrl or siRAD21 and treated with either EtOH or OHT for 48 h. (**a**) Immunofluorescence staining of BrdU incorporation (green) at 48 h post siRNA transfection/treatment. Nuclei were stained with DAPI (in blue). Cells were pulse-labeled with BrdU for 30 min and then fixed for IF. Insets and side panels: magnification of nuclei and their classification based on BrdU incorporation. Cells with widespread nuclear incorporation of BrdU were defined as Pan-BrdU, while cells displaying only clearly identifiable focal BrdU incorporation were defined as BrdU foci. BrdU-foci-positive cells were sub-set based on the foci count, as indicated. Yellow circles highlight the BrdU-foci counted. (**b**) Bar graph of the percentage of BrdU-positive cells, classified as described in (**a**). (**c**) Kinetic analysis of S-phase entry of Nocodazole synchronized cells assessed by continuous BrdU incorporation. The experimental design is outlined in the upper panel. Data are presented as mean±standard deviation (Wilcoxon matched-pairs signed rank test: *P<0.05). Representative images of BrdU immunostaining at 18 h post release are shown on the right. (**d**) Graph of the percentage γH2AX in EdU-positive cells detected at different time points post release from a double thymidine block. Error bar represents S.D. of two independent experiments. The percentage of EdU-positive cells detected at 12 h post the first or the second thymidine release (marked by the black arrows) is indicated next to the symbols (mean value). OHT was added after the second thymidine release. (**e**) Quantification of RPA foci determined by immunofluorescence (48 h post transfection). Left: representative images of foci in RPA-positive nuclei; right: dot-plot, number of cells is indicated within parentheses. Significant differences are indicated by the asterisks (*t*-test: *P<0.05). Effect size (Cohen’s *d*): +OHT (siCtrl *versus* siRAD21)=0.36

The above observations raised the possibility that enforced activation of Myc, while promoting S-phase entry may lead to overt RS. To address this issue, we performed single cell analysis based on Immunofluorescence (IF) detection of bromodeoxyuridine (BrdU) incorporation, in order to score both widespread DNA synthesis (i.e. cells displaying pan-nuclear distribution of BrdU incorporation) and low intensity DNA replication (i.e. cells with a limited number of BrdU foci, suggestive of a low frequency of active forks). Depletion of RAD21 decreased the percentage of cells with pan-nuclear BrdU incorporation, to values that matched those determined by FACS analysis (Figures 1h and 3b). In addition, IF analysis revealed the concomitant increase of cells showing a limited number of BrdU foci (Figures 3a and b). The total count of BrdU-positive cells (pan-positive cells+cells with BrdU foci) scored in RAD21-depleted cells was comparable to the total cell count measured in wild type, suggesting that the S-phase defect of RAD21-depleted cells was due to the low number of active replication forks.

S-phase entry was partially rescued by MycER activation, which led to an increase in the fraction of pan-nuclear BrdU-positive cells and a concomitant reduction of BrdU-foci-positive cells, thus suggesting that Myc, by restoring the expression of S-phase genes, could promote DNA synthesis even in the absence of RAD21 (Figure 3b). Yet, this rescue was partial, suggesting an intrinsic requirement of RAD21, which was beyond the regulation of the expression of genes needed for DNA synthesis. To directly assess Myc ability to drive RAD21-depleted cells into S-phase, we first synchronized RAD21 silenced cells in prometaphase by a Nocodazole block and then, as cells were released from the block, we activated MycER. As expected, MycER activation accelerated S-phase entry in wild-type cells (Figure 3c). While RAD21 depletion in control cells prevented S-phase entry, we observed a progressive increase of cells initiating DNA synthesis when MycER was activated (Figure 3c). This enhancement, albeit lower than control cells, confirmed that enforced Myc activation was able to rescue S-phase entry in cells lacking RAD21.

Given that Myc deregulation may trigger DDR associated to RS,^31^ we wanted to assess whether Myc-induced DDR in siRAD21 cells was triggered during DNA replication. Thus we conducted a kinetic analysis of Myc-induced DDR in cells, previously synchronized at the G1/S boundary by thymidine, which were then released from the G1/S block in the presence of EdU, in order to monitor DNA synthesis. Upon Myc activation, there was a progressive accumulation of γH2AX as cells started DNA synthesis (EdU-positive cells), which was particularly pronounced in cells that were depleted of RAD21 (Figure 3d). This was Myc dependent, since in the absence of Myc activation neither wild-type cells nor siRAD21 cells showed γH2AX positivity (Figure 3d). Also, neither thymidine-arrested cells nor EdU-negative cells displayed γH2AX accumulation (Supplementary Figure 4) upon OHT treatment, thus indicating that Myc-induced DDR in siRAD21 cells occurred during DNA replication. In line with this, quantitative analysis of Replication Protein A (RPA) foci as a marker for RS showed that RPA foci were further augmented by simultaneous Myc activation and RAD21 silencing (Figure 3e). Overall, these data implied that while Myc could promote DNA synthesis in cohesin-depleted cells by supporting those transcriptional programs that are required for S-phase entry, it could not fully rescue DNA replication.

**Figure 4:**
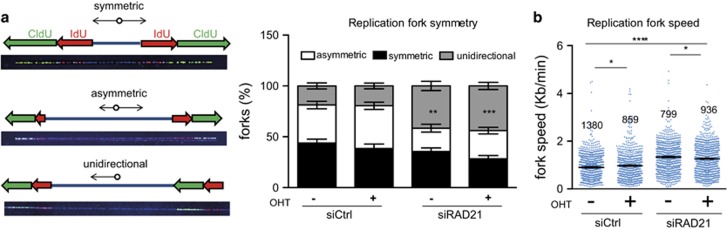
Analysis of DNA replication in RAD21-depleted cells by DNA combing. U2OS-MycER cells were transfected with siCtrl or siRAD21 and treated with OHT or ethanol at the same time. At 48 h post transfection, cells were sequentially pulsed with IdU and CldU for 30 min and then collected for DNA combing. (**a**) Left: Representative images of symmetric, asymmetric and unidirectional replication forks; Right: cumulative bar graph of the analysis of replication fork symmetry. (**b**) Dot-plot of replication fork rates. Number of forks analyzed for each experimental group is indicated above. Significant differences are indicated by the asterisks (*t*-test: *P<0.05; **P<0.01; ***P<0.001; ****P<0.0001). Effect size (Cohen’s *d*): −OHT (siCtrl *versus* siRAD21)=0.78; +OHT (siCtrl *versus* siRAD21)=0.51

To further assess this point, we monitored replication forks progression by DNA combing assay. RAD21 depletion resulted in a dramatic increase in the percentage of unidirectional replication forks (Figure 4a), suggesting that a considerable fraction of replication forks was prematurely terminated in these cells. This was not rescued by Myc activation, thus indicating that while Myc can increase the efficiency of DNA replication, it cannot solve the intrinsic fork defect that follows loss of cohesins. There was also a slight increase in the speed of the replicating forks suggesting that RAD21 may provide a kinetic barrier to DNA synthesis, which may be needed to ensure fidelity in DNA replication (Figure 4b).

### Myc triggers checkpoint activation and G2/M arrest in RAD21-depleted cells

**Figure 5:**
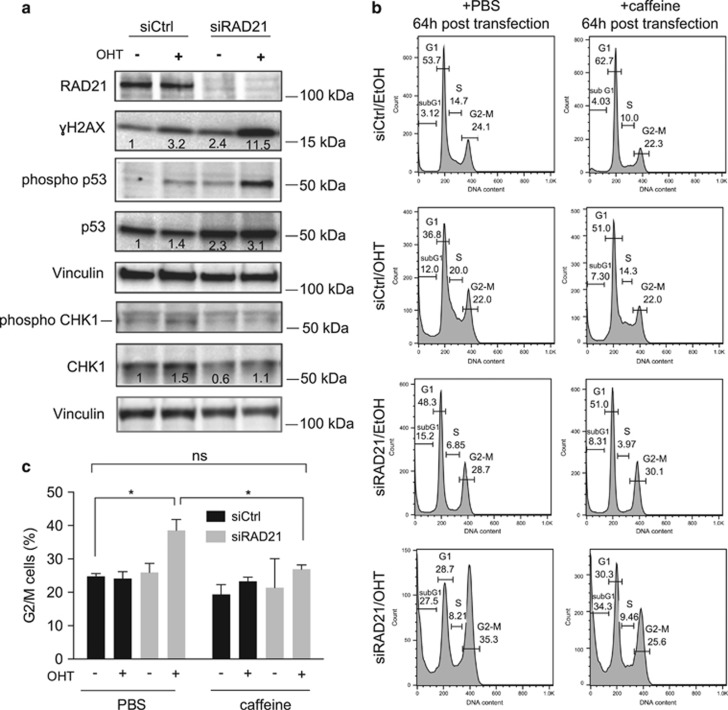
Myc activation triggers DNA damage checkpoint activation in RAD21-depleted cells. (**a**) Western blot analysis of phosphorylated DDR proteins in siRNA transfected cells treated with ethanol or OHT after 48 h. Numbers are the normalized intensity of the bands. (**b**) Cell cycle distribution by FACS analysis, of cells treated with caffeine (5 mM) or PBS (as a control), as indicated. (**c**) Bar graph of the percentage of G2/M cells (*n*=3, *t*-test: *P<0.05; n.s., not significant)

DDR due to fork stalling and collapse is expected to trigger a DNA damage checkpoint response and cell cycle arrest. Indeed, cell cycle distribution of RAD21-depleted cells revealed an increase in the G2/M population following Myc activation (Figure 1g), raising the possibility that the Myc-induced DDR observed in RAD21-silenced cells might engage the G2/M checkpoint. Checkpoint activation was further confirmed by the analysis of p53, the main effector of the G2/M checkpoint. Western blot analysis showed the expected stabilization and phosphorylation of p53 on Ser15, following MycER activation, which reflects Myc intrinsic tumor suppressive activity (Figure 5a). Similarly, silencing of RAD21 led to a slight increase in p53 level and phosphorylation on Ser15, thus suggesting that loss of RAD21 *per se* may lead to low levels of replication stress and checkpoint activation. Relevantly, both p53 levels and its phosphorylation were further increased by MycER activation, thus indicating that combined activation of Myc and loss of RAD21 synergize in the activation of the checkpoint. Given the prominent role of ATM/ATR as apical regulators of the DNA damage checkpoint,^32^ we tested whether their pharmacological inhibition would revert the G2/M arrest observed in RAD21-silenced cells following MycER activation. Treatment with caffeine, an ATM/ATR inhibitor, decreased the percentage of G2/M cells in RAD21-depleted cells where MycER-was activated, to levels comparable to controls (Figures 5b and c). In line with this, shRNA mediated knockdown of p53 in MEFs where RAD21 was silenced and MycER was activated, led to an increase in the fraction of polyploid cells (Supplementary Figure 5) and to the loss of Myc-induced apoptosis (Supplementary Figure 5c), both typical signs of the loss of the p53 checkpoint in fibroblasts. Overall, these results suggest that the ATM/ATR-p53 mediated checkpoint was engaged upon Myc activation in RAD21-depleted cells to control cell cycle arrest and cellular viability.

### Induction of DNA synthesis in RAD21-depleted cells is not a common property of cellular oncogenes

**Figure 6:**
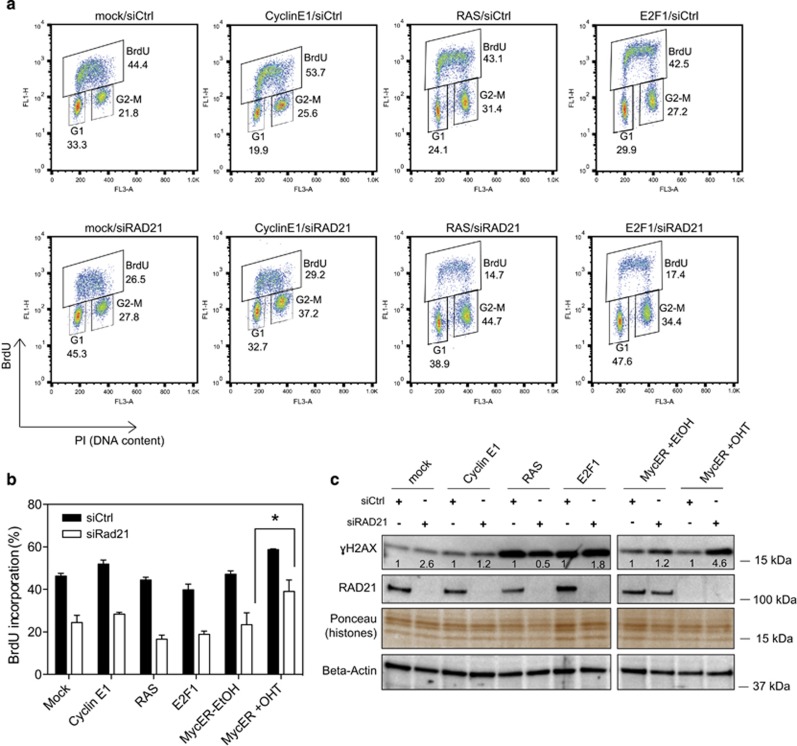
Analysis of DDR and cell cycle distribution upon overexpression of selected oncogenes in RAD21-depleted cells. (**a**) FACS profiles of U2OS cells stably overexpressing Cyclin E1, RAS, E2F1 or empty vector (mock). Cells were pulse-labeled with BrdU before collection. (**b**) Bar graph shows the percentage of BrdU incorporation. Significant differences are indicated by asterisk (two-way ANOVA+Tukey’s multiple comparisons test, *P<0.05). (**c**) Western blot analysis of γH2AX in cells at 48 h post siRNA transfection. RAD21 knockdown by siRNA was confirmed by western blotting. Beta-Actin and total protein stained with Ponceau was used to control for equal loading. Numbers are the normalized intensity of the bands

To investigate whether the genetic interaction linking Myc to RAD21 could be generalized to other oncogenes able to induce replication stress and DDR, we generated stable U2OS cell lines for Cyclin E1, RAS^V12D^ or E2F1. Cell cycle analysis of oncogene-overexpressing cells transfected with siRAD21 revealed a reduction in the S-phase population to an extent similar to mock transfected cells (Figures 6a and b), suggesting that, in contrast to Myc, none of these oncogenes is capable of enforcing DNA replication when RAD21 levels are reduced. Moreover, analysis of the DNA damage marker γH2AX in the oncogene over-expressing cells did not reveal any further increase in its phosphorylation when RAD21 was silenced (Figure 6c) and no signs of overt cell death were observed in these cells (data not shown).

## Discussion

In this work we carried out a genetic dissection of the functional interaction between Myc and the cohesin complex in the regulation of cellular proliferation and cell survival. We focused on two key processes that are controlled by both genes: (i) transcription and (ii) DNA replication.

In our experiments, RAD21 depletion led to alterations in mRNA expression, with a strong effect on the expression of Myc and its target genes. While this is in line with many reports,^13, 15, 22, 23, 33^ it is still unclear to what extent Myc downregulation is due to the direct control of cohesin on Myc transcription or rather secondary to cell cycle arrest. The evidence that Myc was downregulated already at short time points following depletion of RAD21 (i.e. 24 h) suggests a direct control of RAD21 over Myc expression. At later time points, when Myc level was further decreased, both cell cycle and Myc-induced transcription were deeply affected. Importantly, activation of MycER rescued Myc-induced transcription and, in part, cell cycle, thus indicating that (i) Myc-induced transcripts are RAD21 (and cohesin) independent and that (ii) Myc-induced transcriptional programs are sufficient for S-phase entry in cohesin compromised cells.

An important point is that while the restoration of Myc-dependent transcriptional programs was sufficient to drive RAD21-depleted cells into S-phase, DNA synthesis could not be fully rescued. This was due to the essential structural role of cohesins and confirmed that loss of cohesin affects DNA replication possibly by lowering the efficiency of replication fork firing and by impairing their stability, as loss of RAD21 reduced the number of S-phase cells in asynchronous cultures and led to inefficient fork firing. Replicating forks also presented a remarkable increase in unidirectional DNA synthesis, thus revealing a profound defect in bi-directional DNA replication.

This fully accounted for the role of cohesins in the assembly of the pre-replication complex and in the spatial organization of replication units delimited by chromatin loops which are anchored by cohesins.^34, 35^ We also noted that, although DNA replication was globally reduced upon RAD21 depletion, the speed of DNA synthesis was paradoxically higher, an effect possibly stemming from a general relaxation of chromatin organization, which may facilitate DNA replication over short DNA tracks.

While our data confirms previous evidence supporting a role for RAD21 in regulating DNA synthesis,^35^ such study failed to detect impairments in S-phase initiation, replication fork symmetry and DNA synthesis rate. Although understanding the reasons for these discrepancies will require further assessment, we note that this study failed to detect downregulation of Myc target genes such as ORCs and MCMs, suggesting that contrary to what we (this work) and others^22, 26^ have observed, in their experiments silencing of RAD21 did not lead to Myc downregulation. We also note that cells used by Guillou *et al.* (i.e. HeLa cells) may be unusually robust in the control of S-phase, as evidenced by their insensitivity to CDC6 silencing.^35^ This is possibly due to the expression of the HPV-16 proteins E6/E7, viral oncogenes known to promote DNA synthesis.^36^ All of the above may account for the more severe S-phase defects observed in our experiments.

Myc activation, while increasing the efficiency of DNA synthesis, did not rescue the intrinsic fork defects due to loss of RAD21, thus forcing cells into acute replication stress, which involved a strong DDR and the engagement of the replication checkpoint. This accounts for the synthetic lethality we and others have observed^27^ and is in line with published work on Myc-mediated somatic cell reprogramming where RAD21 loss in somatic cells prevented Myc-induced DNA synthesis, thus impairing their heterokaryon-mediated reprogramming.^37^ This dual role of Myc in rescuing S-phase and triggering aberrant DNA replication may be a part of a more general scenario where upstream regulators of DNA replication may play multiple (and sometimes opposite) roles, such as in the case of p21, whose chronic expression in precancerous settings fuels genomic instability by deregulating replication origins’ licensing.^38^

Overall our data point to a regulatory circuit whereby cohesins, by controlling Myc expression, act as a topological checkpoint for S-phase entry that evolved to prevent RS. While further work will be needed to fully understand the origin of such replicative defect, it is interesting to note that, along the genome, cohesin sites are clustered within topologically associating domains which are also early replication units; here cohesins may bookmark transcriptional units during the cell cycle^26^ and provide topological information needed for proficient DNA synthesis, to avoid the interference of gene transcription during DNA replication (Supplementary Figure 6).

Intriguingly, we show that the rescue of DNA synthesis in RAD21-depleted cells is a peculiar property of Myc not shared by other genes or oncogenes known to be potent inducers of mitotic proliferation. This is possibly related to the strong and direct transcriptional control exerted by Myc on cell cycle genes; indeed in many instances other oncogenes control cellular proliferation by engaging Myc.^39^ Importantly, Myc is also a key regulator of nucleotide biosynthesis genes, a peculiar function that is essential to fuel DNA replication and to prevent RS upon oncogene-driven DNA synthesis, thus providing a metabolic checkpoint to S-phase progression.^36^

In this respect, our work may provide a conceptual framework for the pathological mutations of cohesins identified in human cancers. Cohesins are frequently mutated in cancer^40, 41^ and although a thorough assessment of their impact is still ongoing, it is reasonable to predict that in the majority of the cases the mutations identified so far lead to (partial) loss of function.^42, 43^ Given the strong proliferative index of tumor cells, loss of cohesin’s function may lead to RS and DDR, thus fueling cancer evolution by increasing genetic instability of cancer lesions. On the other hand, the role of cohesins in preventing replication stress may account for the observation that Myc and RAD21 are frequently co-amplified in cancer (Supplementary Figure 7); here the intrinsic RS promoted by Myc deregulation may be restrained by the concomitant reinforcement of cohesin-mediated DNA synthesis, thus permitting faithful clonal expansion of tumor cells. We propose that, similarly to the role of the ATR/CHK1 checkpoint,^31^ the cohesin complex, by preventive RS, may represent a liability of Myc driven cancers.

## Materials and methods

### Cell lines, cell culture, cell transduction and transfection

U2OS cells were cultured in DMEM (Lonza, Basel, Switzerland) supplemented with 10% FBS, 1% penicillin/streptomycin (GIBCO, Life Technology, Monza, Italy) and 2 mM L-Glutamine (GIBCO) in 5% CO_2_ incubator. U2OS cells were transduced with pbabe-puro retrovirus encoding c-MycER^28^ or empty backbone. For siRNA transfection, cells were reverse-transfected with Lipofectamine RNAiMAX (Invitrogen by Life Technologies, Monza, Italy) reagent according to the manufacturer’s instruction. Human siRAD21 (siGENOME Human RAD21, SMARTpool M-0006832) and siRLuc (P-002070-01) were purchased from Dharmacon (Lafayette, CO, USA). Mouse siRAD21 oligos (s72658, s72659) and human siRNAs against SMC3 (s17426) and NIPBL (s24589) were purchased from Ambion, Life Technologies (Italy). All siRNAs were used at 25 nM final concentration during transfection. OHT was added in the time of transfection at 400 nM final concentration.

### Immunoblotting

Cells were lysed in an appropriate volume of lysis buffer containing 20 mM Hepes pH 7.5, 0.5 M NaCl, 5 mM EDTA, 10% Glycerol and 1% Triton-X 100, containing protease and phosphatase inhibitors. Protein extract (20–30 *μ*g) was resolved by SDS-PAGE (4–15% gradient precast TGX polyacrylamide gel – Bio-Rad Laboratories, Segrate, Milan, Italy) and followed by standard western blot procedures. The following antibodies were used: anti-phospho-Histone H2AX (Merck Millipore, Vimodrone, Italy, JBW301), anti-RAD21 (sc-54325, Santa Cruz, Heidelberg, Germany), anti-CHK1 (sc-8408, Santa Cruz), anti-CHK1 pS345 (#2348), anti-p53 (sc-1311, Santa Cruz), anti-p53 pS15 (#9286, Cell Signaling, Leiden, Netherlands), anti-Vinculin (V9131, Sigma-Aldrich, Milan, Italy), anti-H3 (Ab1791, Abcam, Cambridge, UK) and anti-c-Myc (ab32072, Abcam). Anti-mouse and anti-rabbit HRP-conjugated secondary Abs were purchased from Sigma-Aldrich. Immunoblots were developed with ECL reagents on BioRad ChemiDoc system and the image was processed using Image Lab 4.0 (Bio-Rad Laboratories, Segrate, Milan, Italy). Numbers within western blotting pictures are the relative intensities of the bands determined by densitometric analysis normalized by both the vinculin signal (loading control) and the indicated reference sample (usually siCntr).

### Immunofluorescence

Immunofluorescence was performed on cells grown on coverslips. For BrdU immunofluorescence, BrdU-pulse labeled cells were first fixed, permeabilized and then treated with DNaseI (100 U/ml, New England Bioscience/NEB, Ipswich, MA, USA) at 37 °C for 30 min prior to incubation with Anti-BrdU (B44, #347580, BD Biosciences, San Jose, CA, USA). For RPA staining, before fixation, cells were subjected to *in situ* cell fractionation as previously described.^44^

### Flow cytometry and cell cycle analysis

Asynchronous growing cells were pulse-labeled with BrdU (100 *μ*M) for 30 min prior to harvesting. After fixation, cells were treated with 1 ml of 2 N HCl for 20 min to expose labeled DNA. HCl was then neutralized with addition of 3 ml of sodium borate (0.1 M, pH 8.5) for 2 min. Cells were then incubated with anti-BrdU antibody (BD Biosciences). Following incubation with secondary fluorescence labeled antibody, cells were resuspended in propidium iodide (2.5 *μ*g/ml) containing RNase A (250 *μ*g/ml) and stored at 4 °C until FACS analysis.

For γH2AX/EdU (5-Ethynyl-2′-deoxyuridine) staining, EdU-pulse labeled cells were first stained with primary mouse anti-phospho H2AX antibody (Millipore, JBW301) followed by incubation with FITC-conjugated anti-mouse secondary antibody. After a washing step, cells were subjected to Click reaction with Alexa fluor 647 alkyne for 30 min using Click-iT kit (Life Technologies) according to the manufacturer’s protocol. Cell pellets were resuspended in 2.5 *μ*g/ml propidium iodide plus 250 *μ*g/ml RNase A and stored at 4 °C until FACS analysis. Cell cycle analysis performed on FACSCalibur flow cytometer using CellQuest software. Flow cytometry data analysis was performed using FlowJo software.

### DNA combing

Asynchronously growing cells were sequentially labeled with 25 *μ*M IdU for 30 min followed by a brief wash with PBS and then 30 min incubation with 200 *μ*M CldU in the cell culture medium. After labeling, cells were trypsinized, harvested and then embedded in agarose plugs until further analysis. The plugs were treated with proteinase K, then DNA was extracted and combed on silanized coverslips. DNA fibers were incubated first with a mouse anti-ssDNA antibody (Chemicon, Millipore, Vimodrone, Italy) followed by Alexa 546 coupled-secondary antibody (Molecular Probes, Invitrogen, Moza, Italy) staining. Incorporation of halogenated nucleotides was detected with specific antibodies (IdU: mouse anti-IdU/BrdU, B44, #347580, BD Biosciences, San Jose, CA, USA; CldU: rat anti-CldU/BrdU, Abcam) and visualized with appropriate secondary antibodies. Images were acquired automatically with a spinning disk confocal microscope, and the individually labeled DNA molecules were manually measured with ImageJ, as previously described.^45^ Fork speed measurements were based on both labels if the CldU replication signals (second label) were flaked by DNA signals. In these instances fork speed was calculated as: (length of CldU signal [green]+length of IdU signal [red])/labeling time. In cases whether the CldU signal was not flanked by DNA, the fork speed was calculated using only the replication signal of the first label (IdU), as (length of IdU signal [red])/labeling time. Forks were scored into three different groups based on their level of symmetry: (i) symmetric fork, when the difference between left and right fork speed was less than 30% (ii) asymmetric fork, in which the difference between left and right fork speed is between 30 to 99% (iii) unidirectional fork, in which the difference between left and right fork speed is 100%, thus only one replication fork departs from a replication origin.

### RNA extraction and analysis

Cells were lysed with QIAzole lysis reagent and total RNA was purified using RNeasy RNA extraction kit (Qiagen, Hilden, Germany) according to the manufacturer’s instruction, including the on-column DNaseI digestion. Extracted and purified RNA was then used for cDNA synthesis with Superscript reverse transcriptase synthesis kit (Invitrogen). Synthesized cDNA was used for subsequent real-time RT-PCR. Quantitative RT-PCR reaction was performed using SYBR PCR master mix (Applied Biosystems, Life Technologies, Monza, Italy) in a BioRad CFX96 system (Bio-Rad Laboratories). Results were normalized to RPLPO expression and were plotted relative to control cells. Data are presented as mean±S.D. The oligos used in real-time PCR are listed below.


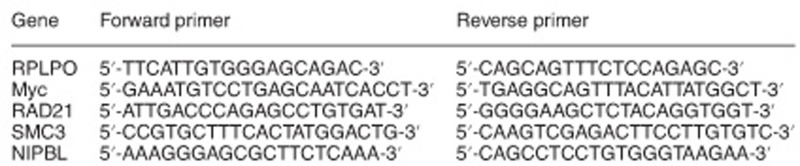


For RNA-seq, 5 *μ*g of purified RNA was first treated with Ribozero rRNA removal kit (Illumina, San Diego, CA, USA) and then precipitated with ethanol. RNA quality and removal of rRNA were checked with the Agilent 2100 Bioanalyser (Agilent Technologies, Santa Clara, CA, USA). RNA-seq was performed on two biological replicates. Libraries for RNA-seq were then prepared with the TruSeq RNA Sample Prep Kit v2 (Illumina) following the manufacturer’s instruction. RNA-seq libraries were sequenced with the Illumina HiSeq 2000 to a read length of 50 bp. Computational analysis of NGS data was performed as described.^46^

### Bioinformatic analyses

RNA-seq NGS reads were sequenced with the Illumina HiSeq2000 platform. Filtering, alignment and differential expression analysis were performed with HTS-flow.^47^ Reads were trimmed or masked if nucleotide quality Q<20. Filtered reads were aligned to the hg19 using TopHat2. The final alignment file was processed for removing PCR duplicates. The number of mapped reads on exons per kilobase of transcript per million mapped reads (exonic reads per kilobase per million, eRPKM) was calculated to rank the expressed genes. Differential expression between control (siCtrl) and treated cells was performed using the Bioconductor package DESeq2. We defined DEGs those with *q*-value<0.05, and log2 fold change>0.5 (upregulated) or log2 fold change<−0.5 (downregulated). Raw (FASTQ) ChIP-seq data for Myc on U2OS-Myc cells were retrieved from the GEO database (accession number GSM1231597). ChIP-seq reads were analyzed with HTS-flow: first, they were aligned with BWA to hg19 genome, then peaks were called using MACS2. ChIP-seq peaks mapping on promoters were defined as 2000 bp upstream and 200 bp downstream from the nearest transcription start site (RefSeq annotation). Genes with at least one peak overlapping with their promoter were considered Myc-bound genes. Intersection of the list of Myc-bound genes and Myc-deregulated genes (genes whose log2FC>0.5 or<−0.5, *q*-value<0.05 in OHT-treated cells *versus* control sample), resulted in Myc-bound-up and Myc-bound-down gene list, respectively. Two way unsupervised hierarchical clustering (Ward algorithm, JMP software, SAS Institute Inc, Cary, NC, USA) was used to identify the two clusters of Myc-rescued genes. Coherence of the clustering was further verified by the correlation analysis and ranked heatmap visualization (Figure 1b).

### Statistical analysis

The bars shown represent mean±s.d. or mean±S.E.M. as indicated. The statistical analyses were performed using either GraphPad Prism or JMP (SAS institute Inc.).

*P*-values were calculated using two tailed *t*-test, unless otherwise stated (*P<0.05, **P<0.01, ***P<0.001 and ****P<0.0001).
