# Supplementary material for: The cohesin complex prevents Myc-induced replication stress
Source: Cell Death Dis. 2017 Jul 27;8(7):e2956–. doi: 10.1038/cddis.2017.345 (PMC5550886; doi:10.1038/cddis.2017.345)
Supplement: Supplementary Figures [file cddis2017345x1.pdf]

**Supplementary Figure 1**

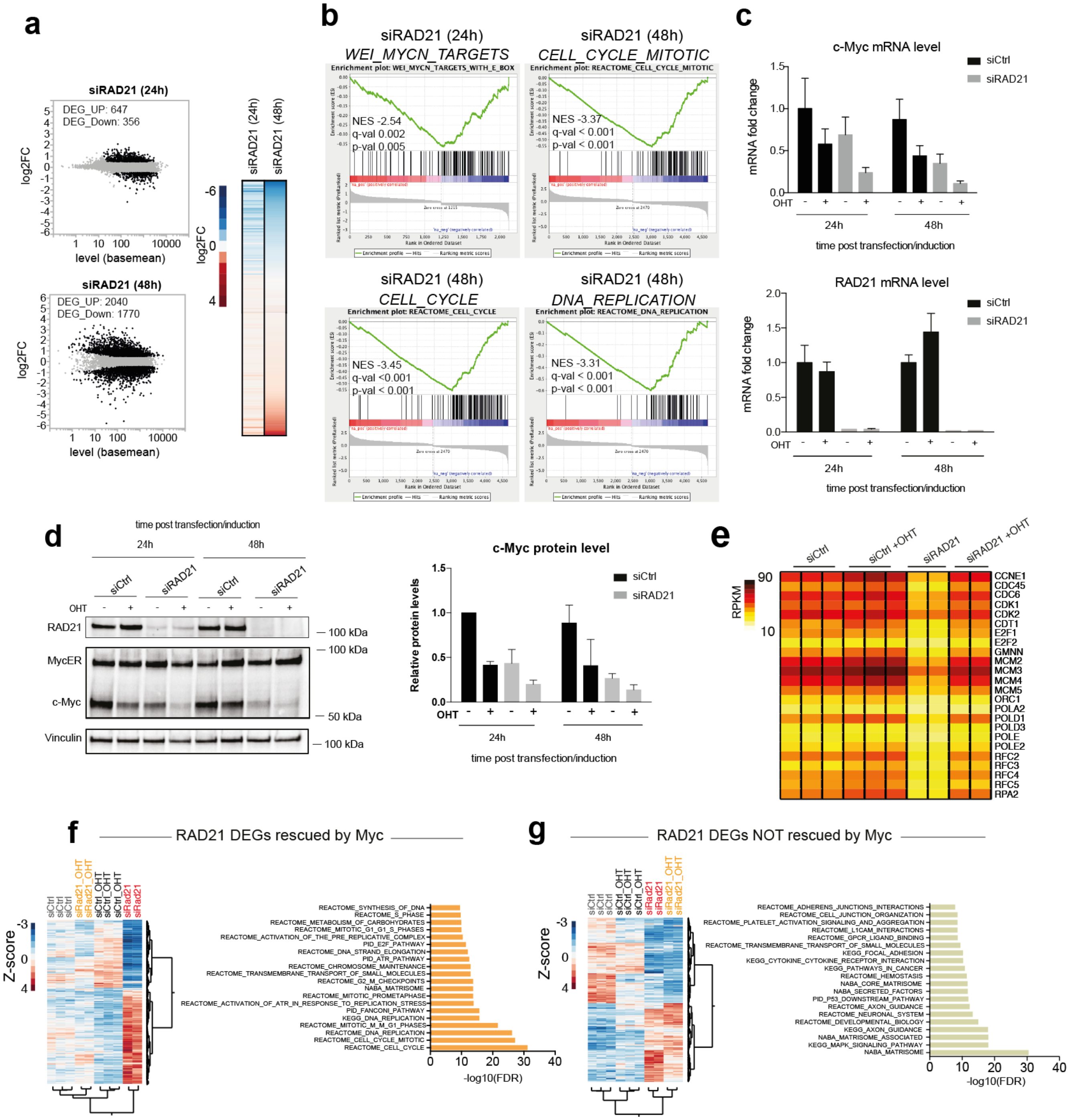


**Supplementary Figure 1.** RAD21 knock down alters the expression of Myc but does not affect its transcriptional activity.

U2OS-MycER cells were transiently transfected with siRNAs targeting either RAD21 (siRAD21) or Renilla Luciferase (siCtrl) and were simultaneously treated with either OHT (to activate MycER) or ethanol (as mock activation). (a) Scatter plots of siRAD21 transfected

cells at 24 (top) and 48 (bottom) hours post transfection. The y-axis shows the log2 fold change in gene expression in RAD21-depleted cells relative to control cells (siCtrl). The x-axis shows RNA expression levels of each gene expressed as the mean read count (baseMean). Each dot represents a gene, with black dots representing genes with significantly altered expression (adjusted p value < 0.05). The number of up- and down-regulated genes is shown in each graph. On the right, ranked heat-map showing the log2 fold change (Log2FC, relative to siCtrl) of the differentially expressed genes identified in siRAD21-transfected cells at 24 and 48 hours post transfection. Red and blue colors indicate up- and down-regulated genes, respectively. (b) Representative gene set enrichment analysis of differentially expressed genes identified at 24 or 48 hours post RAD21 silencing. (c) Quantitative RT-PCR analysis of c-Myc (top) and RAD21 (bottom) expression level in cells transfected with siRAD21 or siCtrl (in the presence or absence of OHT) at 24 and 48 hours after transfection. mRNA expression level was normalized with a housekeeper gene RPLPO and is plotted relative to control cells (siCtrl + EtOH at 24h). (d) Western blot analysis of Myc, showing MycER protein (upper band) and endogenous Myc protein (lower band). RAD21 knockdown by siRNA was confirmed by western blotting at 24 and 48 hours after transfection. Vinculin was used as a loading control. On the right, bar graph of the densitometric analysis of the western blot, showing endogenous Myc protein level normalized to vinculin and relative to the control sample (siCtrl+EtOH at 24h). (e) Heat-map of the expression level of a representative subset of genes linked to DNA synthesis. (f) Left: Hierarchical clustering of RAD21-DEGs rescued by Myc (data was Z-score normalized); right: top 20 gene sets identified by GSEA. (g) Left: Hierarchical clustering of RAD21-DEGs not rescued by Myc (data was Z-score normalized); right: top 20 gene sets identified by GSEA.

## Supplementary Figure 2

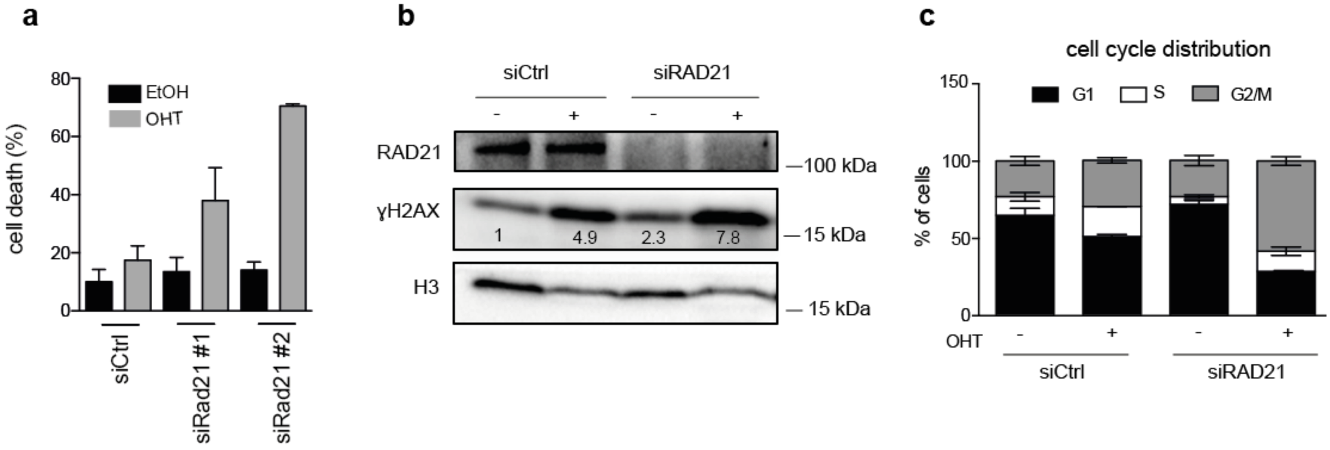


**Supplementary Figure 2**. The effect of RAD21 depletion in mouse embryonic fibroblasts (MEFs). R26-MycER immortalized MEF cells were transfected with two different siRNAs against RAD21 (siRAD21 #1 and #2) or a non-targeting siRNA (siCtrl) and treated with OHT or ethanol in the same time. (a) Percentage of cell death, measured by trypan blue staining at 48 hours after transfection. (b) Western blot analysis for H2AX phosphorylation in transfected cells at 48 hours. Numbers are the normalized intensity of the ɣH2AX band. (c) Percentage of cells in each phase of cell cycle at 48 hours post transfection, as determined by FACS analysis on propidium iodide-stained cells.

## Supplementary Figure 3

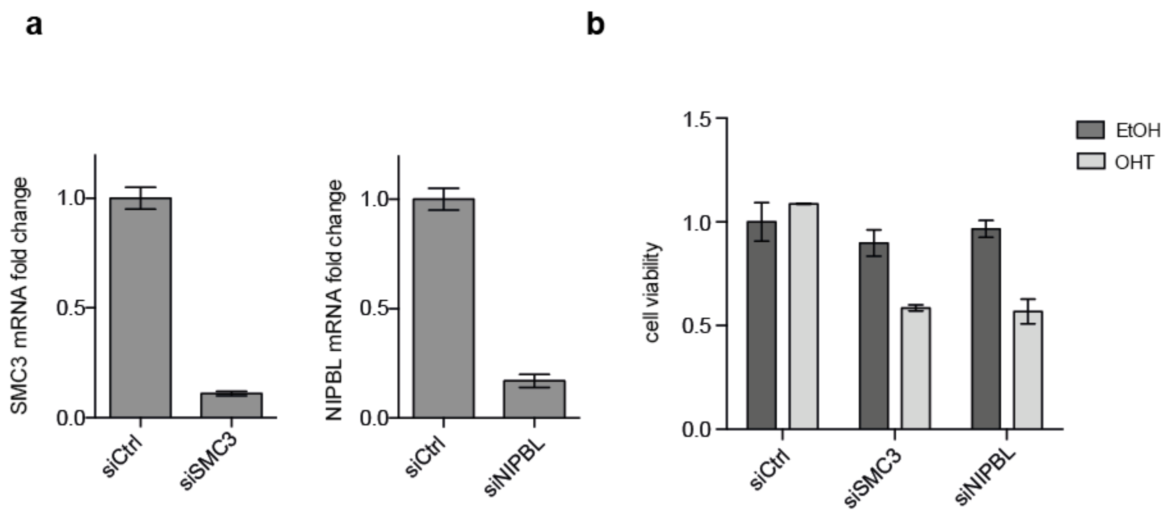


**Supplementary Figure 3**. The effect of Myc activation on cell viability of cohesin deficient U2OS cells. U2OS-MycER cells were transfected with siRNAs targeting SMC3 or NIPBL or siCtrl and treated with ethanol or OHT at the same time. (a) Bar graphs show relative mRNA expression levels of SMC3 (left) and NIPBL (right) in transfected cells at 48 hours compared to siCtrl-transfected cells. (b) Relative cell viability measured by MTT assay, at 48 hours post transfection and MycER activation.

## Supplementary Figure S4

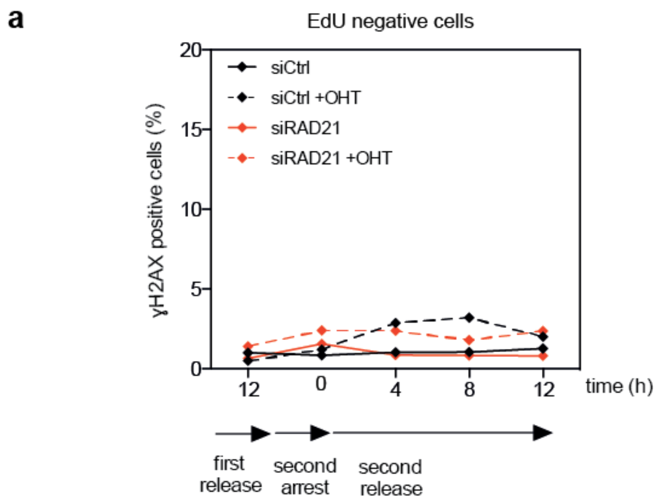


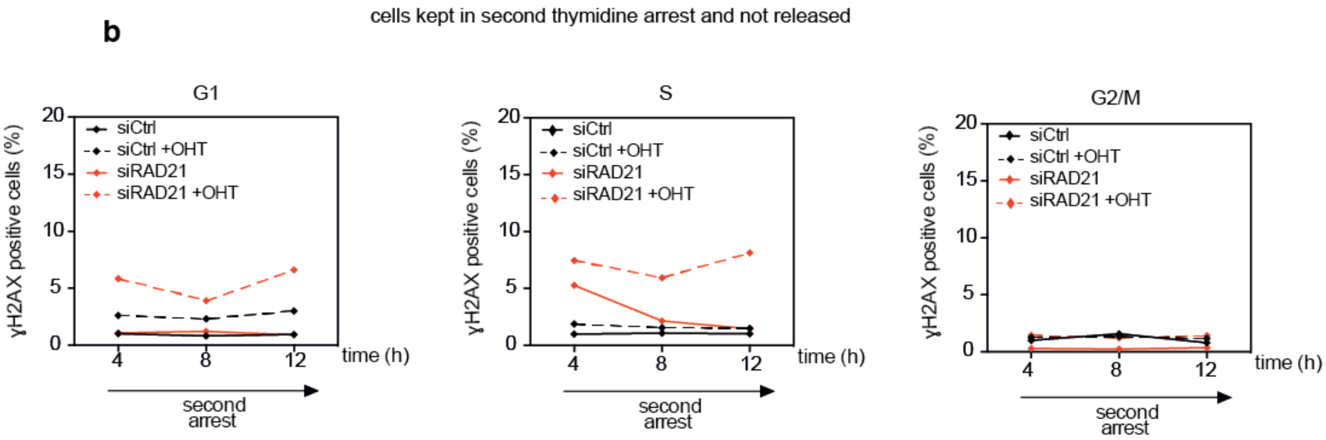


**Supplementary Figure 4.** FACS analysis of γH2AX in thymidine-synchronized cells. U2OS-MycER cells were transfected with siRAD21 or siCtrl and subjected to two rounds of thymidine arrest and release as explained in Figure 3d. (a) Quantification of γH2AX positive cells in EdU-negative population in released cells relative to control cells (siCtrl). (b) Quantification of γH2AX in thymidine-arrested cells in each phase of cell cycle over time relative to control sample. Cell cycle phases were determined based on DNA content.

## Supplementary Figure S5

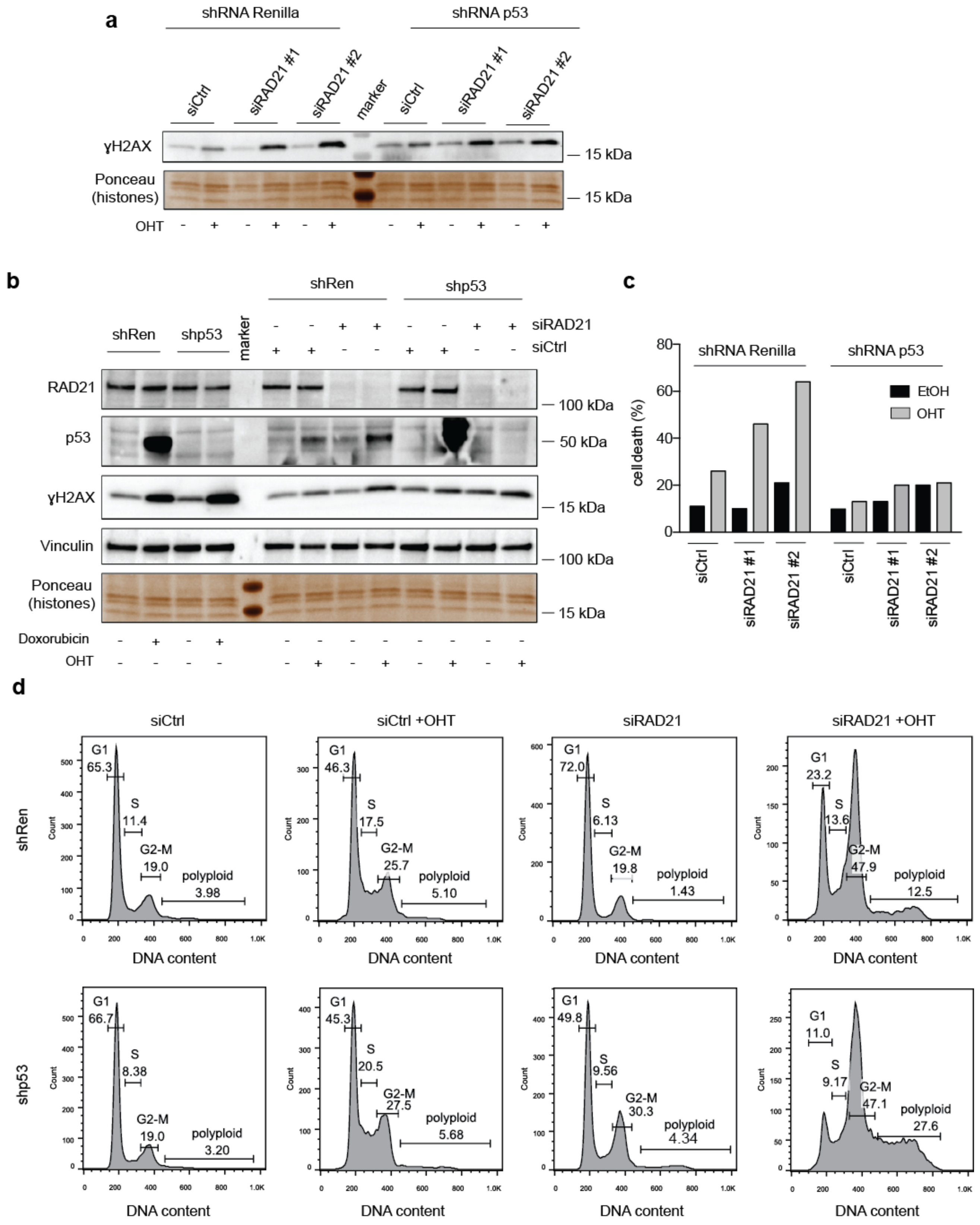

**Supplementary Figure 5.** The impact of RAD21 depletion on the p53-mediated checkpoint activation. R26-MycER MEFs (infected with shp53 or shRenilla) were transfected with siRAD21 or siCtrl and simultaneously treated with OHT or ethanol. Cells were harvested after 48 hours of transfection for further analyses. (a) Western blot analysis of H2AX phosphorylation in transfected cells with two different siRNAs against RAD21 (siRAD21 #1 and #2). (b) Western blot analysis for p53 accumulation in transfected cells. The efficiency of p53 knockdown was evaluated by detecting the accumulation of stabilized p53 upon doxorubicin treatment (1 μM for 16h). Vinculin and ponceau staining for histones were used as loading controls. (c) Percentage of cell death in siRNA transfected cells measured by trypan blue staining. (d) Cell cycle profile of transfected cells as determined by propidium iodide staining and FACS analysis.

## Supplementary Figure S6

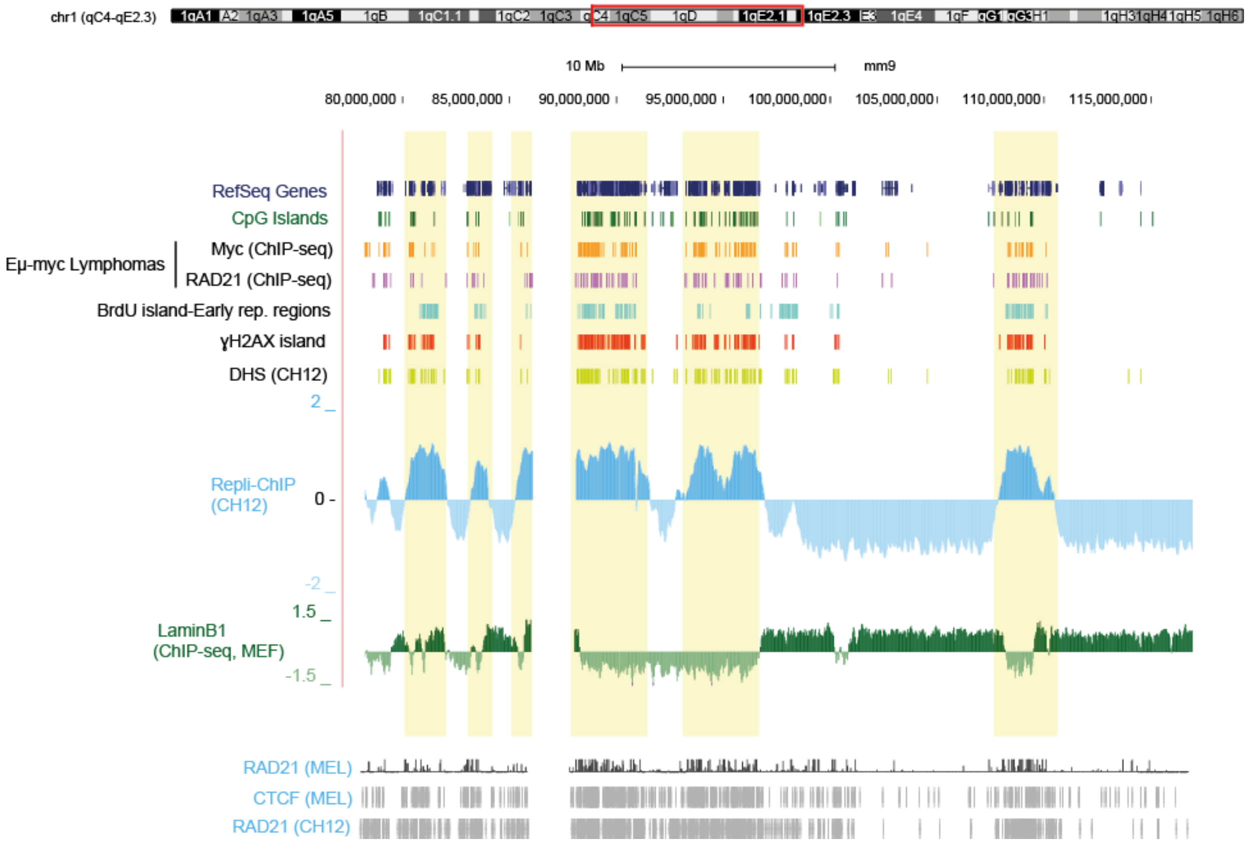


**Supplementary Figure 6.** Genome wide distribution of cohesin binding.

Genome browser snapshot of murine Chromosome 1 showing how early replicating regions defined by both repli-ChIP and BrdU islands[1] (region highlighted in yellow) define euchromatic islands (low LaminB1 binding) that are enriched for transcribed genes, Myc and RAD21 binding. γH2AX island marks region of recurrent replicative stress[1].

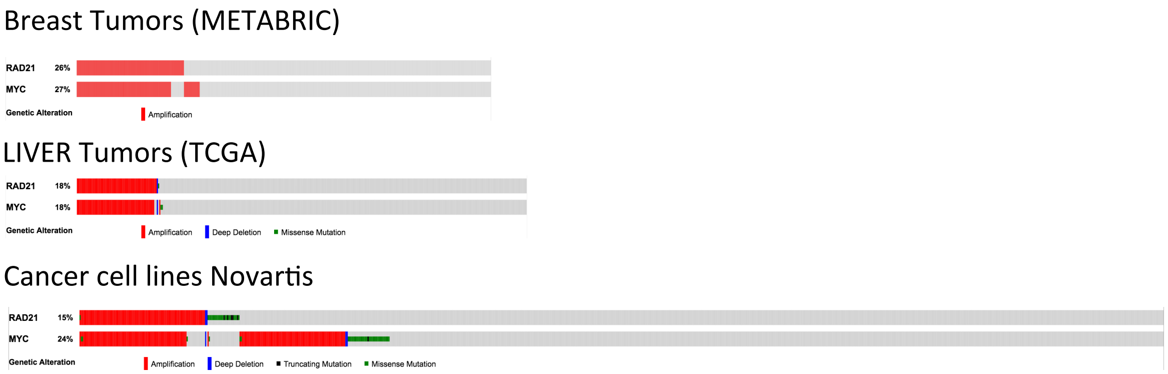


**Supplementary Figure 7.** Co-amplification of MYC and RAD21 in tumors.

TCGA data was analysed using the cBioPortal (http://www.cbioportal.org/index.do)[2]

Supplementary References

1 Barlow, J. H. *et al.* Identification of early replicating fragile sites that contribute to genome instability. *Cell* **152**, 620-632, doi:10.1016/j.cell.2013.01.006 (2013).

2 Cerami, E. *et al.* The cBio cancer genomics portal: an open platform for exploring multidimensional cancer genomics data. *Cancer Discov* **2**, 401-404, doi:10.1158/2159-8290.CD-12-0095 (2012).
