# Supplementary material for: The cohesin complex prevents Myc-induced replication stress
Source: Cell Death Dis. 2017 Jul 27;8(7):e2956–. doi: 10.1038/cddis.2017.345 (PMC5550886; doi:10.1038/cddis.2017.345)
Supplement: Supplementary Tables legends [file cddis2017345x6.docx]

Supplementary Tables legends

**Supplementary table 1.**

GSEA analysis of differentially expressed genes (DEGs) identified in U2OS-MycER cells upon Myc activation and RAD21 silencing.

**Supplementary table 2.**

GSEA analysis of RAD21 regulated genes sub-setted as either rescued or not-rescued by MycER activation.

**Supplementary table 3.**

List of differentially expressed genes (DEGs) identified in U2OS-MycER cells.

**Supplementary table 4.**

List of MycER regulated genes bound or not by Myc at their promoter.
